# Distribution of two isoforms of tryptophan hydroxylase in the brain of rainbow trout (*Oncorhynchus mykiss*). An in situ hybridization study

**DOI:** 10.1007/s00429-021-02322-8

**Published:** 2021-07-02

**Authors:** Mauro Chivite, Esther Leal, Jesús M. Míguez, Jose Miguel Cerdá-Reverter

**Affiliations:** 1 Laboratorio de Fisioloxía Animal, Departamento de Bioloxía Funcional e Ciencias da Saúde, Facultade de Bioloxía and Centro de Investigación Mariña, Universidade de Vigo, 36310 Vigo, Spain; 2 Food Intake Control Group, Departamento de Fisiología y Biotecnología de Peces, Instituto de Acuicultura de Torre de la Sal, Consejo Superior de Investigaciones Científicas (IATS-CSIC), 12595 Castellón, Spain

**Keywords:** Trout, Teleost fish, Brain, Serotonin, TPH, In situ hybridization

## Abstract

Serotonin (5-HT) is one of the principal neurotransmitters in the nervous system of vertebrates. It is initially synthesized by hydroxylation of tryptophan (Trp) by means of tryptophan hydroxylase or TPH which is the rate-limiting enzyme in the production of 5-HT. In most vertebrates, there are two isoforms of TPH present, TPH1 and TPH2, which exhibit different catalytic or substrate specificity as well as different expression domains. Studies carried out in mammals show that only *tph2* is expressed in the brain whereas *tph1*-mRNA is primarily localized in the enterochromaffin cells and pineal gland. A large number of neurons are also considered to be serotonergic or “pseudo-serotonergic” as they accumulate and release 5-HT yet do not produce it as no amine-synthetic enzymes are expressed, yet a combination of 5-HT transporters is observed. Therefore, *tph* expression is considered to be the only specific marker of 5-HT-producing neurons that can discriminate true 5-HT from pseudo-serotonergic neurons. This work examined in situ hybridization to study the mRNA distribution of one paralogue for *tph1* and *tph2* in the central nervous system of rainbow trout. Results show a segregated expression for both paralogues that predominantly match previous immunocytochemical studies. This study thus adds valuable information to the scarce analyses focusing on the central distribution of the expression of serotonergic markers, particularly *tphs*, in the vertebrate brain thus characterizing the true serotonergic brain territories.

**Supplementary Information:**

The online version contains supplementary material available at 10.1007/s00429-021-02322-8.

## Introduction

The monoamine serotonin or 5-hydroxytryptamine (5-HT) is one of the major neurotransmitters of the central nervous system (CNS). The 5-HT metabolic pathway is initiated by tryptophan (Trp) being hydroxylated to the intermediate metabolite 5-hydroxytryptophan (5-HTP) by the action of the tryptophan hydroxylase (TPH). 5-HTP is subsequently decarboxylated to become 5-HT by the aromatic L-amino acid decarboxylase (AADH) enzyme. Due to the rapid activity of AADH, 5-HTP levels are usually low, making TPH the rate-limiting enzyme in the 5-HT production (Höglund et al. 2019). In the pineal gland, 5-HT is further processed by the serial action of the aryl-alkylamine *N*-acetyltransferase (AANAT) and hydroxyindole-*O*-methyltransferase (HIOMT) to produce melatonin (Falcón et al. 2010). Following synthesis, 5-HT is accumulated in intracellular organelles, the synaptic vesicles in neurons, by the vesicular monoamine transporters (VMAT1 and VMAT2) (Gaspar and Lillesaar 2012). The 5-HT transporter SERT or SLC6A4 also mediates the reuptake from the synaptic cleft back into the presynaptic boutons thus finalizing 5-HT effects and allowing neurotransmitter recycling by presynaptic neurons (Rudnick and Sandtner 2019; Bader 2020). Catabolism of 5-HT to 5-hydroxyindol acetic acid (5-HIAA) is mediated by the consecutive action of the monoamine oxidases (MAO-A and MAO-B) and the aldehyde dehydrogenase (ALDH2) (Höglund et al. 2019). A large number of cells/neurons could also be regarded as serotonergic or “pseudo-serotonergic” as they accumulate and release 5-HT yet do not produce it as no amine-synthetic enzymes are expressed, yet a combination of 5-HT transport is observed (Gaspar and Lillesaar 2012).

Therefore, *tph* expression is specific for the 5-HT producing cells/neurons and the only specific marker of 5-HT-producing neurons as *aadh* and *mao* are also expressed by other monoaminergic neurons, many cells use SERT/SLC6A4 for 5-HT reuptake without undergoing synthesis (Norton et al. 2008; Lillesaar 2011). TPH appears in two isoforms, TPH1 and TPH2. In mammals, peripheral organs such as the enterochromaffin cells, mammary and pineal glands, placenta and pancreatic beta cells predominantly use TPH1 for 5-HT synthesis. On the contrary, TPH2 is basically a central isoform, yet some cells in the periphery also use it for 5-HT synthesis, such as the serotonergic myenteric cells (Panula et al. 2010; Gaspar and Lillesaar 2012).

Neurons containing 5-HT have been identified in all major metazoan groups suggesting an early appearance of the system during animal evolution (Cornide-Petronio et al. 2013). The central serotonergic system has been characterized in depth in all vertebrate classes (Sako et al. 1986; Van Mier et al. 1986; Adrio et al. 1999; Hay-Schmidt 2000; Manger et al. 2002; Lillesaar and Gaspar 2018; Lozano et al. 2020) yet most studies have used specific antibodies to map serotonergic neurons. In mammalian species, central 5-HT is confined within the raphe nuclei brainstem where *tph2* is expressed. On the contrary, in non-placental vertebrates the central serotonergic system disseminates to other brain structures of the forebrain which include pretectal and hypothalamic areas (Lillesaar 2011; Gaspar and Lillesaar 2012; Lozano et al. 2020; Timothy and Forlano 2020). However, the absence of studies using reliable serotonergic markers, *tph1* and *tph2* expression (Lillesaar 2011), makes it unclear whether extra raphe 5-HT neuronal populations are truly serotonergic (producing 5-HT) or pseudo-serotonergic (storing 5-HT).

Distribution of 5-HT immunoreactivity in the diencephalon and mesencephalon of rainbow trout (*Onchorhynchus mykiss*) was reported in the early 80’s revealing six 5-HT immunoreactive areas (Frankenhuis-van den Heuvel and Nieuwenhuys 1984) yet expression studies to elucidate the true 5-HT neurons in the trout brain have never been conducted. Similar to mammalian models and other teleost fish, the involvement of central serotonergic pathways in key aspects of the trout physiology and behaviour, including the regulation of food intake (Ruibal et al. 2002; Pérez-Maceira et al. 2014), stress response and coping styles (Lepage et al. 2002; Øverli et al. 2005; Schjolden et al. 2006; Gesto et al. 2015), cognitive function (Carpenter and Summers 2009; Vindas et al. 2014), and aggression (Winberg et al. 2001; Øverli et al. 2004; Lepage et al. 2005) has been reported. Studies carried out in a variety of fish species have also demonstrated the involvement of the central serotonergic system in reproduction (Prasad et al. 2015), sleep regulation (Oikonomou et al. 2019), locomotion (Gabriel et al. 2009), fear and anxiety (Egan et al. 2009), neurogenesis (Kuscha et al. 2012; Pérez et al. 2013) and neuronal regeneration (Sobrino-Cameán et al. 2019). Therefore, the study of serotonergic neurons in rainbow trout gains a general importance in understanding this wide array of roles played by 5-HT. This paper characterizes the expression of *tph1* and *tph2* in the brain of rainbow trout showing a segregated expression for both isoforms that greatly match previous immunocytochemical studies (Frankenhuis-van den Heuvel and Nieuwenhuys 1984). Our experiments add valuable information to the scarce analyses focusing on serotonergic marker expression, particularly *tphs* in vertebrates, which are basically limited to pigeons and zebrafish (Gaspar and Lillesaar 2012).

## Materials and methods

### Phylogenetic analysis

Inference of *tph* evolutionary relationships was performed using protein data sets of complete coding sequences, which excluded the hagfish *Eptatretus burgueri* (111 amino acid sequences), from 39 sequenced genomes (Esembl, http://www.ensembl.org/index.html). Multiple sequence alignments were generated using ClustalX 2.1 on the whole number of species, only fish species or exclusively salmonid sequences and the evolutionary history was inferred using maximum likelihood, minimum evolution, maximum parsimony and neighbour-joining methods on the JTT matrix-based model using MEGA. A phylogenetic view of thp evolutionary relationships was obtained by MEGA 7.02.21, moreover, cladograms and robustness were estimated at each branching node by 100 random bootstrap replications.

### Fish and tissue processing

Two-year-old rainbow trout (*n* = 10; ≈ 200 g) were obtained from a local fish farm (Aigua Natura dels Ports, Tarragona). The specimens were anesthetized in 2-phenoxyethanol 0.02% v/v (Sigma), transcardial perfusion was carried out using 50 ml of physiological saline solution (NaCl 0.65%), and subsequently specimens were perfused with the same volume of fixative containing paraformaldehyde (PAF; 4%) in phosphate buffer (PB; 0.1 M, pH = 7.4). Following decapitation, the brains were removed, postfixed overnight in the same fixative at 4 °C, dehydrated, and embedded in Paraplast (Sherwood, St. Louis, MO, USA). Serial 6-µm cross sections were cut using a rotary microtome. One section every 200-µm was mounted on 3-aminopropyltriethoxylane-treated (TESPA) slides and then air-dried at room temperature overnight. Six consecutive series, covering the length of the rainbow trout brain, were made, one of these series was stained with cresyl-violet 0.1% (Cerdá-Reverter et al. 2001) for detailed identification of brain nuclei and the remaining series were used for hybridization with sense and antisense probes. The sections were stored at 4 °C under dry conditions and used for hybridization within 2 weeks.

### Synthesis of riboprobes

Total mRNA was extracted from the fish brain using Trizol reagent (Life Technologies, Grand Island, NY, USA) and treated with RQ1-DNAse (Promega, Madison, WI, USA). One μg of the total RNA was reverse transcribed using Superscript II reverse transcriptase (Promega) and random hexamer primers (Promega) in 20 μl final volume. A pull of the cDNA obtained was subsequently used as a template for PCR amplification with Taq DNA polymerase (Promega) using specific oligoprimers for *tph1a* (fw: 5′-CAAGATCGACGAGAACAAGGACA-3′; rv: 5′-GTGAACTCGATATGCGGAATTGG-3′; 528 bp) and *tph2* (fw: 5′-CCTGTTCTTGAAAGAGACGTCTG-3′; rv: 5′-CCAGGGTCAAACATCTTCACTGAG-3′; 417 bp). PCR fragments were separated onto 1% agarose gel, then purified using NucleoSpin^®^ Gel and PCR Clean-up (Machery-Nagel). Subsequently, the fragments were cloned using pGEM-T easy vector (Promega). Plasmid DNA were obtained using QIAprep Spin Miniprep Kit (Quiagen) and fragments were sequenced on both strands to verify their identity. Clones were linearized with *Sal* I and *Sac* II, respectively, and transcribed for the riboprobes using SP6/T7 RNA polymerase (Promega) and digoxigenin (DIG)-labelled UTPs (Roche). The probes were then treated with RQ1-DNAse-RNAse free (Promega) for 15 min at 37 °C to remove the DNA template. Finally, the probes were purified using Micro Bio-Spin Chromatography Columns (BioRad) and quantified in a Thermo Scientific Nanodrop 2000c spectrophotometer.

### In situ hybridization

Brain slides were deparaffinized, re-hydrated, post-fixed (PAF 4%), and then treated with Proteinase-K solution (20 μg/ml in 50 mM Tris–HCl, 5 mM EDTA at pH 8) for 6 min at RT. Brain slides were washed in PB, post-fixed again in PAF4% for 5 min, rinsed in sterile water, and acetylated in a triethanolamine (0.1 M, pH 8)/acetic anhydride solution for 15 min in constant agitation. Anti-sense or sense cRNA probes of *tph1a* or *tph2* were preheated at 75 °C for 7 min and diluted in hybridization buffer [50% formamide, 300 mM NaCl, 20 mM Tris–HCl (pH 8), 5 mM EDTA (pH 8), 10% dextran sulphate (Sigma), and 1× Denhardt’s solution (Sigma)] at a concentration of 3 ng/µl. Sections were covered with 80–100 μl of hybridization solution and incubated in a humidification chamber at 65 °C O/N. The optimal probe concentration, wash times and hybridization temperature were determined in previous pilot experiments.

Slides were incubated in 5× standard saline citrate buffer (SSC, 150 mM NaCl, 15 mM sodium citrate at pH = 7) for 30 min at 55 °C to remove coverslips. The slides were then rinsed in 2× SSC and 50% formamide for 15 min at 65 °C and immersed in NTE buffer (500 mM NaCl, 10 mM Tris–HCl, 5 mM EDTA, pH 7.5) three times for 5 min at 37 °C. Following the ribonuclease A treatment (40 μg/ml ribonuclease A in NTE) for 15 min at 37 °C, slides were incubated in NTE buffer for 5 min at 37 °C, once in 2× SSC and 50% formamide for 10 min at 65 °C, once in 2× SSC for 10 min at RT and twice in 0.1× SSC for 10 min at RT. Before being incubated with anti-DIG antibody, slides were rinsed 3 times in MAB (150 mM NaCl, 100 mM maleic acid, pH 7.5) containing 40 mg/ml Tween 20 (Sigma) for 5 min at RT and incubated in blocking buffer (150 ml MAB, 150 µl Tween 20, 750 µl normal goat serum (NGS), 75 mg levamisol and 3 g blocking reagent (Roche Diagnostic)) for 3 h. Slides were incubated at 4 °C O/N with primary antibody 1:2000 anti-digoxigenin in MAB plus TWEEN 20. The antibody was removed by washing 6 times in MABT and twice in developing buffer (100 mM Tris, 100 mM NaCl, 50 mM MgCl_2_, pH 9.5) for 10 min at RT. Subsequently, the slides were incubated with chromogen substrates NBT/BCIP (Roche Diagnostic) to develop the staining. Sections were mounted with a mount quick aqueous medium (Bio-Optica) and visualized on an Olympus BX41. Serial sections were stained with 0.1% cresyl violet (Sigma) for cytoarchitectonic analysis. Nissl staining permanently dyes genetic material (DNA and RNA) therefore it is not restricted to neurons exclusively. Anatomical locations were confirmed by reference to a brain atlas of rainbow trout (Billard and Peter 1982) but nomenclature followed (Wullimann et al. 1996).

## Results

### Phylogenetic analysis

Maximum likelihood phylogenetic trees showing the evolutionary relationship of tph proteins for salmonid species, fish species and animal species are shown in Supplementary Fig. 1. The different phylogenetic methods efficiently determine the segregation of vertebrate tph sequences into two main clades for tph1 and tph2, respectively (Supplementary Fig. 1A–C). The genome of most basal phyla has only one *tph* gene, this also occurs in the lamprey (*Petromyzon marinus*) and myxine (*Eptatretus burgueri*) species. However, the genome of the chondrichthyes (*Callorhinchus milii*) displays both *tph1* and *tph2* thus suggesting that the duplication of *tph* genes took place in the gnathostomata following the divergence of the cyclostomata species (Supplementary Fig. 1A, B). Accordingly, tph2 sequence of the chondrichthyes is basal to all tph2 sequences. Tetrapod tph2 sequences (except *Xenopus tropicalis*) are grouped in a common clade which also includes the coelacanth sequence. The second clade includes all tph2 from teleost species with the non-teleost spotted gar (*Lepisosteus oculatus*) as a basal sequence. Lamprey and myxine *tph* seem to be more similar to *tph1* than *tph2* thus suggesting *tph1* was used as a template for gene duplication following the gnathostome divergence (Supplementary Fig. 1A).

Bootstrap values indicated that the phylogenetic methods applied cannot discriminate tph1 sequences as consistently as for *tph2* gene (Supplementary Fig. 1A, B). Significantly, chondrichthyes, lepisosteiforms, coelacanthiforms and tetrapod genomes exhibit only a *tph1* gene suggesting that the presence of *tph1* and *tph2* genes is the ancestral vertebrate condition. As a result of teleost genome-specific duplication (TGSD), most teleost fish exhibit two tph1 genes that had initially been labelled as *tph1a* and *tph1b* yet *tph2* duplication is not found in any species (Xu et al. 2019). *tph1a* and *tph1b* do not form monophyletic groups thus making the evolutionary inferences challenging (Supplementary Fig. 1A, B). Salmonids have undergone an additional duplication by reaching a tetraploid condition, therefore more gene copies are expected including tph1a1/tph1a2, tph1b1/tph1b2, and tph2a/tph2b. In silico data from genome sequencing projects demonstrate that salmonid species exhibit 4 *tph* genes yet a single copy of *tph2* gene (Supplementary Fig. 1A–C). All three other copies are grouped together with fish *tph1a* yet low bootstrap values validate such association (Supplementary Fig. 1A, B). Independent analysis of salmonid tph sequences displays segregated clades for *thp1* and *tph2* (Supplementary Fig. 1C). Furthermore, tph1 sequences are arranged in two subclades suggesting that tph1 forms can be split up into tph1a/tph1b as well as tph1a1/tph1a2. Tph1b/tph1a2 subclade also exhibits two subdivisions/clades which seldom include one sequence of each species, suggesting that two loci for tph1b/tph1a2 are identifiable.

### *Tph1* expression in the rainbow trout brain

Hybridization with sense *tph1a*-cRNA and *tph2*-cRNA probes never generated specific signals in the rainbow trout brain (data not shown) supporting the probe specificity. It should be considered that in situ hybridization cannot discriminate the cell type expressing the specific mRNA, however, up to our knowledge, *tph* expression in the brain is restricted to neuronal cell bodies as no expression has been reported in glia cells including ependymal cells and tanycytes (Perez et al. 2013). Therefore, *tph*-expressing cells in the brain will be referred as neuron from here on. More detailed studies would involve double labelling experiments with glial fibrillary acidic protein (GFAP) to identify potential glia cells expressing *tph*.

**Fig. 1:**
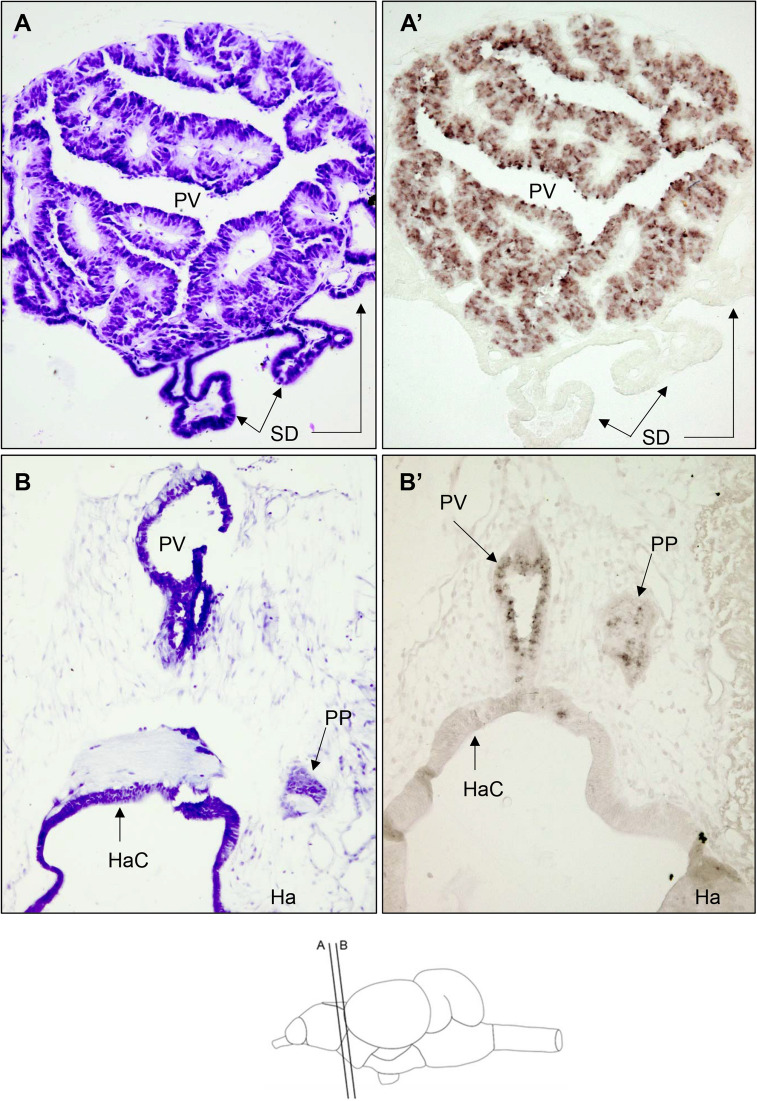
Bright-field photomicrographs of transverse sections of the rainbow trout brain at the level of rostral diencephalon showing *tph1*-expressing cells. Section levels are shown in the schematic drawing at the bottom of the figure. **A** and **B** are Nissl staining with cresyl violet of transverse sections at similar rostrocaudal level of those shown in **A’** and **B’**, respectively. **A’** displays a high expression level in most cells of the pineal vesicle (PV), presumably photoreceptors (see “Discussion” for details), whereas B’ shows lower expression levels in the parapineal organ (PP). The identity of *thp*-expressing cells in the PP remains unknown (see “Discussion” for details). Arrows indicate the *saccus dorsalis* (SD) A and A’, PP, PV and habenular commissure (HaC) in **B** and **B’**. Ha habenula. Scale bar = 50 μm

*tph1* mRNA was massively expressed in the pineal vesicle (PV) of rainbow trout which rostrally appears on the top of olfactory bulbs progressing to the intersection between the telencephalic hemispheres and optic tectum where epithalamus habenula is located (Fig. 1A). At the caudal end of the PV, just dorsal to the habenula and located on only one hemisphere, a conspicuous group of cells conforming to the parapineal organ (PP) also produce *tph1* mRNA (Fig. 1B). Our experiments cannot discriminate the cell type expressing *tph* mRNA in the PV and PP but as discussed later (see “Discussion”) these cells should be photoreceptors.

**Fig. 2:**
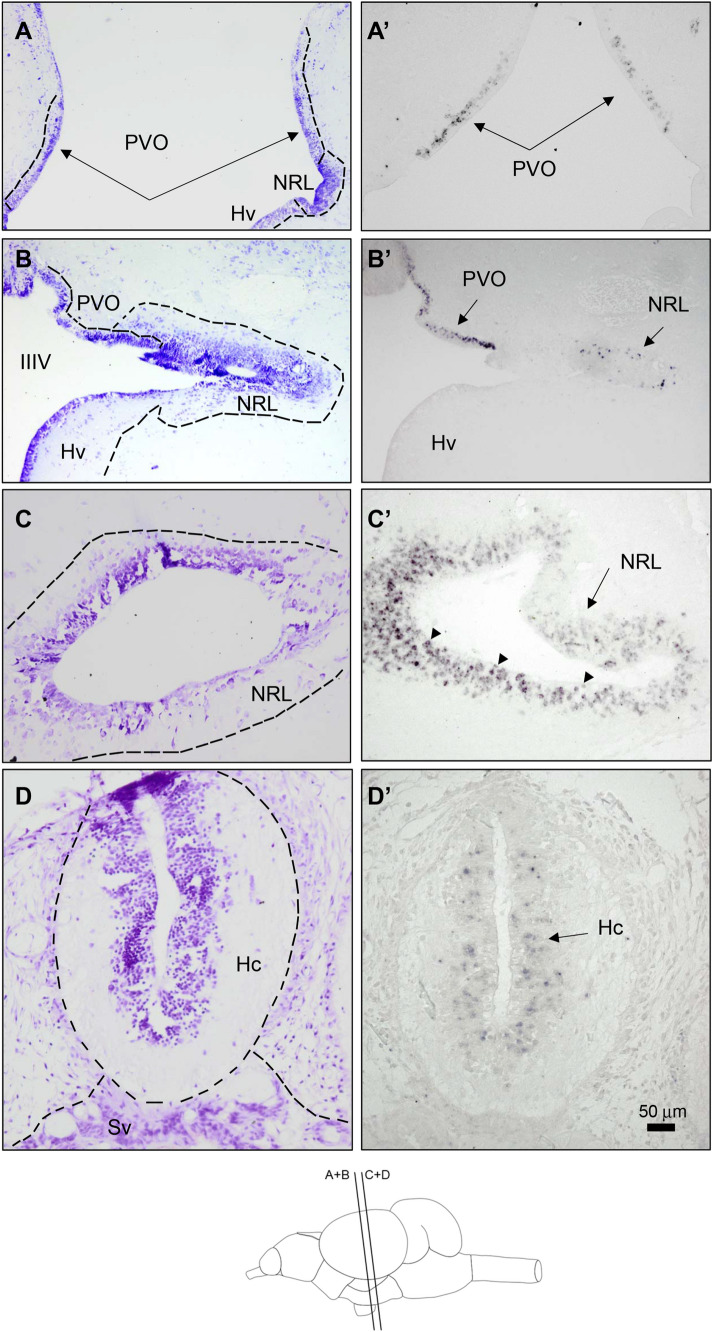
Bright field photomicrographs of transverse sections of the rainbow trout brain showing *tph1*-expressing neurons at the level of rostral (**A**, **A’**, **B**, **B’**) and caudal hypothalamus (**C**, **C’**, **D**, **D’**). Rostro caudal levels of the sections are shown in the schematic drawing at the bottom of the figure. **A**–**D** Provide morphological details by Nissl staining with cresyl violet of transverse sections at a similar level of those shown in **A’**–**D’**, respectively. **A’** Positive *tph1*-expressing neurons in the paraventricular organ of the posterior tubercle (PVO). Arrows in **A** and **A’** show cell (**A**) and *tph1*-expressing neurons in PVO, respectively. **B’** *tph*-mRNA expressing neurons are also present in the lateral opening of the III ventricle (IIIV) at the lateral recess nucleus (NRL) according to Cerdá-Reverter’s (2000) nomenclature or Hd according to Wullimann et al. (1996). Arrows in **B’** show *tph1*-expressing neurons in the PVO and NRL. **C’** Numerous small and rounded positive cells coating the lateral recess. Arrows in **C’** indicate *tph1*-expressions neurons in the NRL, respectively. Arrowheads in **C’** indicate neurons contacting the ventricular wall in the NRL. **D’** Positive cells in the most caudal region of the tuberal hypothalamus (Hc). Arrows in **D’** show positive neurons in the caudal hypothalamus. Scale bar = 50 μm

Closer to the caudal, *tph1* expression in the paraventricular organ (PVO) was found. *Tph1*-expressing neurons in the rostral PVO line the third ventricle accurately coinciding with the lateral aperture of the hypothalamic medial tuberal ventricle (Fig. 2A) which being slightly more caudal will come in contact with the lateral recesses in the inferior hypothalamic lobe. Such neurons seem to migrate initially into the dorsal region of the lateral recesses (Fig. 2B) and subsequently coat the entire perimeter of the hypothalamic lateral recess (Fig. 2C). Also concurring with the lateral expansion of the third ventricle, some *tph1*-expressing neurons coat the ventral hypothalamic region of the third ventricle (Hv) (data not shown). At the caudal pole on the tuberal hypothalamus, the *thp1*-expressing neurons coat the caudal region of the third ventricle in the caudal hypothalamus (Hc, Fig. 2D). Some *tph1*-expressing periventricular neurons in the PVO and NRL (see arrowheads in Fig. 2A, C) appeared to make contact with the ventricular wall thus suggesting a physical link with the cerebrospinal fluid (CSF).

### *Tph2* expression in the rainbow trout brain

**Fig. 3:**
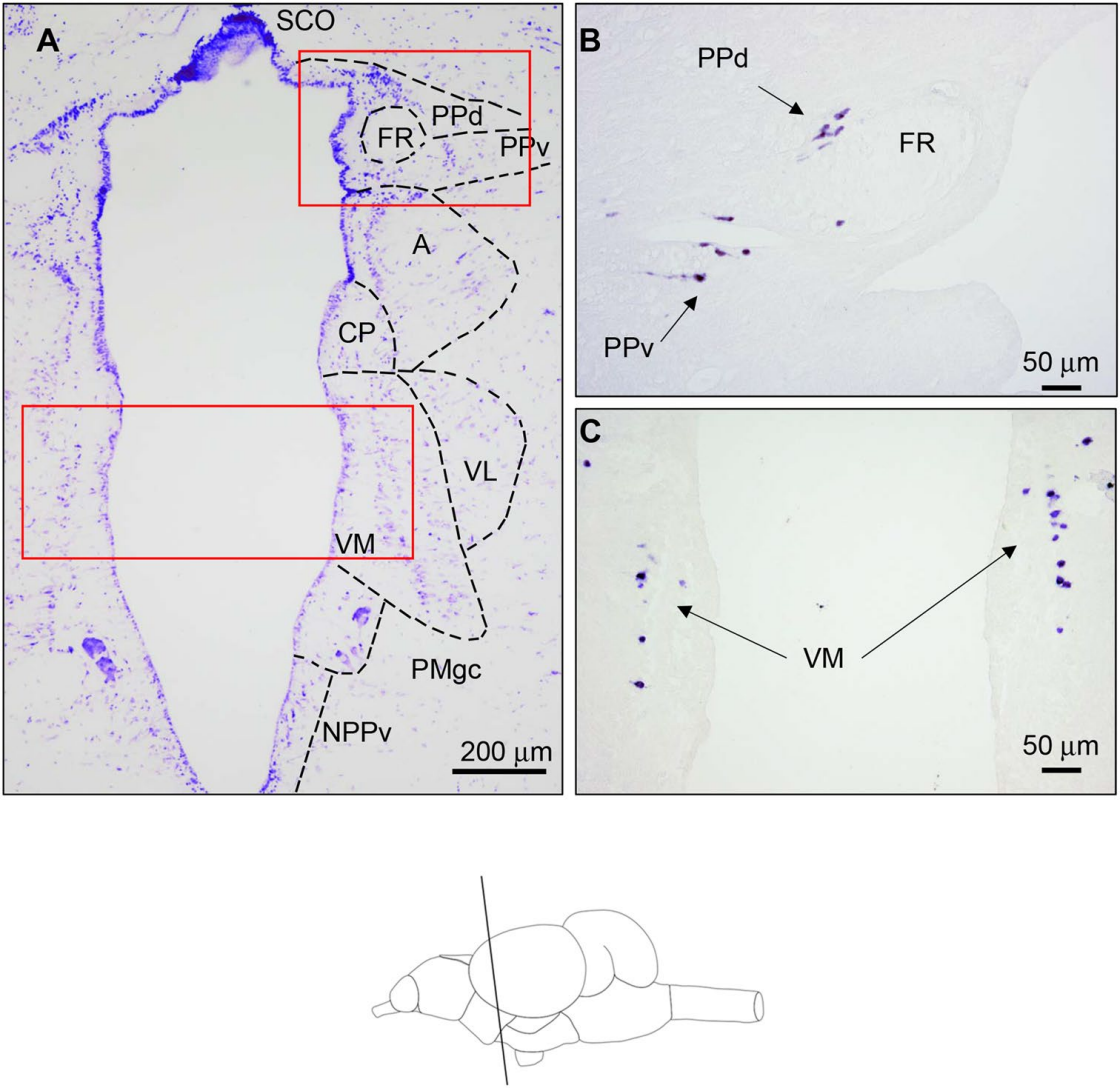
Bright-field photomicrographs of transverse sections of the rainbow trout brain showing *tph2*-expressing neurons at the level of ventral thalamus and pretectal area. Rostro caudal levels of the sections are shown in the schematic drawing at the bottom of the figure. **A** Transverse section stained with cresyl violet that shows anatomical details of a similar section level as in **B** and **C**. Dashed lines in **A** set the limits of different diencephalic and pretectal nuclei after Nissl staining. Red rectangular frames indicate equivalent regions shown in **B** (opposite hemisphere) and **C** (both hemispheres). **B** *tph2*-expressing neurons in the rostral pretectal area at the level of *retroflexus* fascicle (FR) in the dorsal (d) and ventral (v) parts of the pretectal periventricular nucleus (PPd and PPv). **C** Positive neurons in the ventromedial nucleus of the ventral thalamus (VM), note that these cells are organized into a single vertical column. Arrows in **B** (pretectal/synencephalic) and **C** (thalamic) indicate neuronal cell bodies expressing *tph2* mRNA. *tph* expression in the brain is restricted to neuronal cell bodies as no expression has been reported in glia cells including ependymal cells and tanycytes (Perez et al. 2013). A (anterior nucleus of the ventral thalamus), CP (central posterior nucleus on the ventral thalamus), NPPv (posterior periventricular nucleus), PMgc, gigantocellular part of the magnocellular preoptic nucleus, SCO subcommissural organ. Scale bar = 50 μm (**B**) and = 200 μm (**A**, **C**)

**Fig. 4:**
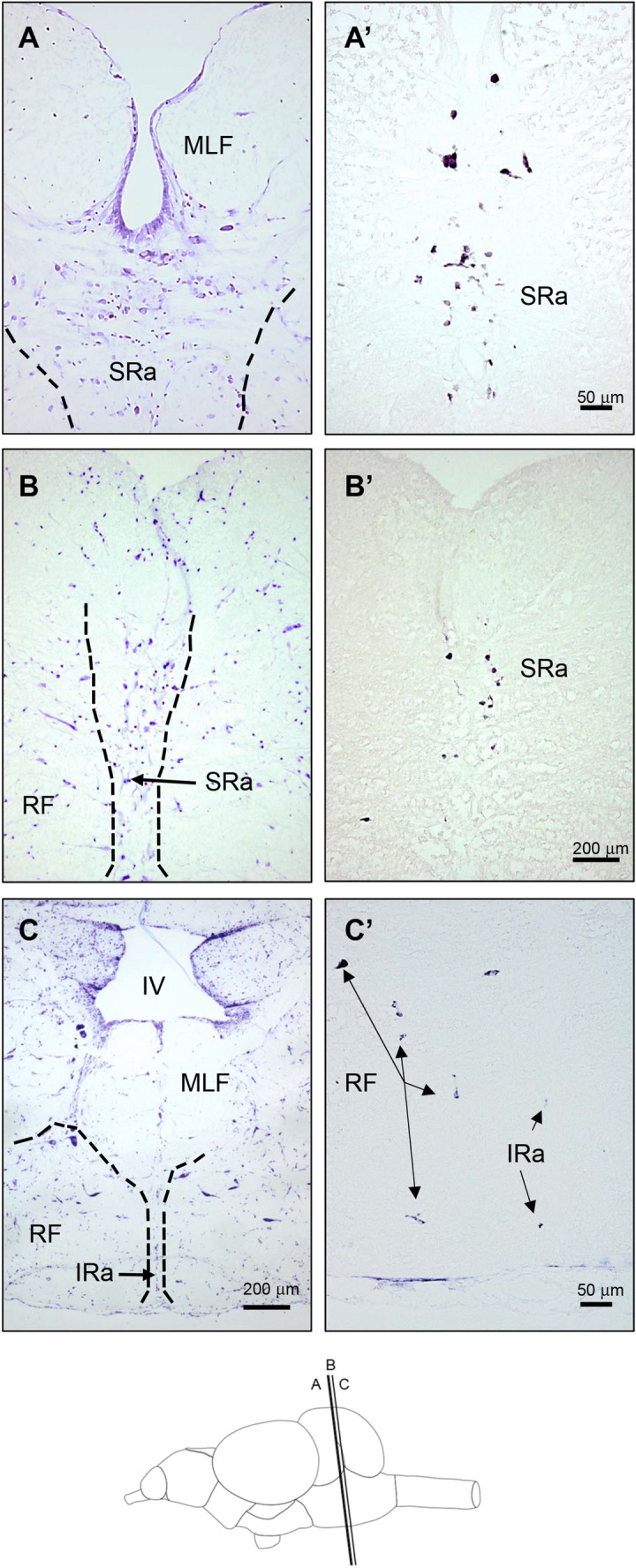
Bright-field photomicrographs of transverse sections of the rainbow trout brain showing *tph2*-expressing neurons at the level of raphe. Rostro caudal levels of the sections are shown in the schematic drawing at the bottom of the figure. **A**–**C** Transverse sections stained with cresyl violet showing anatomical details of a similar section level as in **A’**–**C’**. Dashed lines in **A**–**C** demarcate different nuclei in the posterior brain after Nissl staining. *tph2* expression in the rostral (**A’**) and more caudal (**B’**) superior raphe (SRa). **C’** Arrows indicate disperse positive neurons weakly stained in the inferior raphe (IRa) and the reticular formation (RF). MLF Medial longitudinal fascicle, IV fourth ventricle. Scale bar = 50 μm (**A**, **A’**, **C’**) and = 200 μm (**B**, **B’**, **C**)

*tph2*-expressing neurons were confined to the pretectal area, ventral thalamus and posterior brain (Figs. 3, 4). In the pretectal area, tph2 expression was found in two adjacent neuronal populations dorsally and ventrally surrounding the fasciculus retroflexus (FR), in the so-called dorsal (PPd) and ventral (PPv) periventricular pretectal nucleus (Fig. 3B). The PPd lies immediately caudal to habenula, dorsal to the anterior thalamic nucleus (A), and lateroventral to the subcommissural organ SCO (Fig. 3A). Slightly caudal and around the medioventral and ventral areas of the FR, the PPv begins. At the caudal end of the posterior commissure, both PPd and PPv move laterally away from the ventricle. Tph-mRNA-expressing neurons in the PPd and PPv exhibit fusiform shape and show laterally directed dendritic processes (Fig. 3B). Slightly ventral, yet found at the same rostro-caudal level, a prominent *tph2*-expressing neuronal population is localized in the ventromedial nucleus of the ventral thalamus (VM). thp2-mRNA neurons in the VM are disposed in parallel to the medial ventricular wall and arranged into a single dorsoventral 1-cell thick column (Fig. 3C). *tph2*-expressing neurons are found in the superior raphe (SRa) caudal to the level of the interpeduncular nucleus (Fig. 4). Rostrally, tph2-expressing large fusiform and highly stained round neurons are intermingled with ventrally localized smaller round neurons which are disposed along the medial line (Fig. 4A). Slightly more caudal, the dorsal population of larger tph2-expressing neurons disappears and only smaller neurons placed on the midline remain (Fig. 4B). However, both populations coexist along the rostral pole of the SRa suggesting that they could form differentiated parts of the SRa. In the most caudal region where tph2 expression was detected, some sparse and minute *tph2*-expressing neurons are found in the midline at the level of the inferior raphe (IRa). In the same section, some scattered neurons in the superior part of the reticular formation (RF) are densely stained. Neuronal bodies expressing tph2 in the RF display an ovoid or fusiform shape with long dendritic processes.

## Discussion

Teleost fish have undergone an extra genome duplication commonly known as teleost-specific genome duplication (TSGD) resulting in an extra-duplication of all their genes (Amores et al. 1998). Following this event, many duplicated genes became pseudogenes (loss of function) yet others experienced a process of neofunctionalization, thus acquiring new functions or subfunctionalization in which both copies share the original function. This is also true for the *tph* system. Most vertebrates exhibit two paralogues called *tph1* and *tph2*. However, a single gene copy is found in invertebrates and cyclostomata species thus suggesting that the duplication of ancestral *tph* took place following the divergence of the Gnathostomata (Cornide-Petronio et al. 2013). Lamprey *tph* seems to exhibit more homology to the *tph1* than *tph2 genes*, it is thus conceivable that the *tph1* gene was the substrate for gene duplication. As a result of TSGD, teleost additional *tph* copies are expected, at least *tph1a* and *b* and *tph2a* and *b*, but only a single copy of tph2 is present in all fish species. This suggests that the second form of *tph2* was rapidly pseudogenized following TSGD. On the contrary, most teleost fish exhibit paralogues of *thp1*, such as *tph1a* and *tph1b* (Xu et al. 2019) despite the fact that the conventional phylogenetic analysis could not group them into monophyletic groups. By increasing the genome complexity of teleost fish, some species, including salmonids and cyprinids, have experienced an additional genome duplication resulting in an extra genome tetraploidization. Therefore, salmonids are expected to show two *tph2* paralogues (*thp2a* and *tph2b*) and four paralogues for *tph1* (*tph1a1*, *tph1a2*, *tph1b1* and *tph1b2*). A second form of tph2 could not be found in any tetraploidized teleost species suggesting additional pseudogenization events. From a functional point of view, it is extraordinary how the evolutionary process has systematically deleted any paralogue of the *tph2* gene. Remarkably, the *thp1* system seems to be much more permissive to the presence of additional copies. The salmonid species also exhibit additional copies of *tph1* gene. All salmonid genomes show three *tph1* genes. Phylogenetic relationships (Xu et al. 2019; and present results) and synteny studies (Xu et al. 2019) suggest the presence of *tph1a*, *tph1b1* and *tph1b2* in salmonid fish, therefore, the paralogue of *tph1a* was once more pseudogenized during the evolutionary process. Alignment and phylogenetic studies indicate that the cloned sequences matched *tph2* and *tph1b2* (data not shown), however, henceforth we will use the nomenclature *tph1* and *tph2* to name the cloned rainbow *tph* sequences used in our in situ hybridization studies.

Both members of the TPH family (TPH1 and TPH2) exhibit different catalytic or substrate specificity (Walther and Bader 2003) yet distinct expression domains. In non-tetrapod vertebrates, both isoforms are expressed in specific areas of the CNS. However, in mammalian species, *tph1* expression is restricted to peripheral tissues whereas *tph2* is expressed primarily in the CNS. The lamprey genome only exhibits a *tph* copy which is phylogenetically more related to *tph1* than *tph2*. Since lamprey *tph1* is expressed in both the diencephalic nuclei and pineal gland, the restricted expression of *tph1* in mammalian species seems to be a derived condition (Cornide-Petronio et al. 2013).

The main *tph1* expression levels are found predominantly in the pineal gland and the results observed in rainbow trout effectively verify data reported in other vertebrate species (Bellipanni et al. 2002; Teraoka et al. 2004; Gaspar and Lillesaar 2012). Fish pineal complex consists of the pineal and parapineal organ and the *saccus dorsalis*. The pineal organ consists of the pineal vesicle dorsally located to the telencephalic hemispheres and connected to the brain by a slim pineal stalk (Birba et al. 2014; Rincón Camacho et al. 2016). In rainbow trout, *tph1* is mainly expressed in the pineal vesicle that exhibits three type of cells, i.e. photoreceptor, projection neurons and interstitial cells (Shainer et al. 2017). Only photoreceptor express aralkylamine *N*-acetyltransferase (AANAT), the step-limiting enzyme in the melatonin biosynthetic pathway, therefore, they should produce tph1 to be able to synthesize melatonin as reported in zebrafish (Teraoka et al. 2004). However, it is also plausible that some projection neurons can synthesize 5-HT but this assumption requires further investigation using double labelling with FoxD3/HuC for pineal neurons and GFAP for glia cells. Our results also revealed some expression level in the parapineal organ. The function of this organ remains unknown but it has been shown to project unilaterally to the left habenula in zebrafish (Turner et al. 2016). Some authors have suggested the presence of photoreceptors (García-Fernández et al. 1997) although others were unable to detect cone or rod opsin immunoreactivity (Rincón Camacho et al. 2016). The potential photosensitivity of the parapineal organ cannot be neglected as some other types of photoreceptors could be present (Birba et al. 2014). Our data suggest that the parapineal organ exhibits true serotonergic cells that are potentially able to synthesize melatonin thus further suggesting that it could participate in the regulation of circadian functions through melatonin secretion. Studies showing aralkylamine *N*-acetyltransferase (AANAT) expression, which is the step-limiting enzyme in the melatonin biosynthetic pathway in the parapineal organ, could help to elucidate its participation in the melatonin synthesis.

Previous studies reported 5-HT immunoreactivity in six different areas of the rainbow trout brain (Frankenhuis-van den Heuvel and Niewenhuys, (1984). Our findings complete these earlier studies by characterizing the type of *tph* expressed in the different serotonergic areas. Studies using 5-HT antibodies cannot entirely discriminate between 5-HT accumulating and/or synthesizing neurons (Gaspar and Lillesaar 2012) as *tph* expression is the only specific marker of 5-HT-producing neurons (see “Introduction”). Therefore, our results complement studies by Frankenhuis-van den Heuvel and Niewenhuys, (1984) by discriminating the areas of *tph*-expressing neurons among those showing immunoreactivity to 5-HT. The neurons expressing *tph* will be able to synthesize the amine whereas pseudo-serotonergic neurons will only accumulate the neurotransmitter.

The phenotype of these pseudo-serotonergic neurons is regulated by the transitory expression of 5-HT transporters (SERT and/or VMAT) that promote 5-HT capture which can be retrogradely transported to neuronal perikarya. These neurons do not synthesize the amine, only take it up, thus explaining the low levels of 5-HT that occasionally make difficult their visualization. However, the low staining levels can establish also morphological differences between both serotonergic phenotypes (Lebrand et al. 1996). The functional implications of these pseudo-serotonergic neurons remains uncertain. It has been shown in rodents that cortical fibers with thalamic origin transiently express SERT and VMAT2 to capture 5-HT synthesized in the raphe (Lebrand et al. 1996). This transient pseudo-serotonergic phenotype is only perceptible at postnatal day 1 (P1) in mice pups and abruptly disappears at P10 (Fujimiya et al. 1986; D’amato et al. 1987) coinciding with the absence of SERT expression. The captured 5-HT could serve as an intracellular signal-regulating gene expression in the thalamic neurons or alternatively could regulate thalamic neurotransmission preventing receptor overstimulation during some developmental phases by controlling extracellular levels of the amine. Finally, thalamic neurons could capture and release themselves 5-HT as a borrowed neurotransmitter (Lebrand et al. 1996; Hansson et al. 1998). Therefore, 5-HT of the brain stem could take advantage of existing neuronal networks during particular developmental phases without the need to establish a new neuronal pattern to regulate, for example, the ingrowth and/or axon arborisation.

In the rainbow trout brain, *tph1* expression was detected in the paraventricular organ of the posterior tubercle (PVO). *tph1*-expressing neurons in PVO emerge from the medial region just above the lateral tuberal nucleus and migrate laterally on the dorsal region of the lateral recess to entirely coat the perimeter of the ventricle. *Tph1*-mRNA neurons also cover the medial region on the third ventricle in its most caudal area, also called posterior recess nucleus or caudal hypothalamus. Some authors denominate all these areas of the posterior tubercle as PVO (Pérez et al. 2013; Lozano et al. 2020) by observing three rostro-caudal regions such as anterior, intermediate and posterior (Pérez et al. 2013) regions whereas others also include hypothalamic subdivisions as dorsal and caudal hypothalamus (Timothy and Forlano 2020). Regardless, 5-HT immunoreactivity in this area has been reported in all fish species examined (Lillesaar 2011; Gaspar and Lillesaar 2012) including rainbow trout (Frankenhuis-van den Heuvel and Nieuwenhuys 1984). Positive neurons expressing *tph1* in the PVO of the rainbow trout correspond to the 5-HT immunoreactive neurons previously described in “area 2” by Frankenhuis-van den Heuvel and Niewenhuys (1984), who also described a conspicuous group of immunoreactive neurons in the ventral hypothalamus or nucleus tuberis inferior (nti) (according to Niewenhuys’ nomenclature) denominated “area 3” (Frankenhuis-van den Heuvel and Nieuwenhuys 1984). Only some *tph1*-mRNA expressing neurons were labelled in this “area 3”. Serotonergic neurons coating the posterior hypothalamic recess would correspond to the “area 4” of 5-HT immunoreactive neurons previously described by Frankenhuis-van den Heuvel and Niewenhuys (1984). Remarkably, authors reported only a few neurons surrounding the lateral recess in the inferior hypothalamic lobe, however, results show a profuse *tph1* expression predominantly in the rostral extension of the recess. There is no explanation for this discrepancy other than the fact that the 5-HT synthesized in the lateral recess nucleus is rapidly transported to other areas of the brain or into the ventricular CSF. PVO is a region which is rich in radial glial cells (RGCs) expressing brain aromatase that give birth to 5-HT neurons that come in contact with CSF but also migrate to other regions of the zebrafish brain. The somata of the 5-HT neurons in the PVO are located closer to the ventricle than those of RGCs that extend processes to form a continuous barrier along the ventricular surface. In turn, 5-HT neurons contact the CSF via processes that cross this barrier through small pores (Pérez et al. 2013). In adult zebrafish treated with TPH inhibitors, the number of proliferating cells in the PVO decrease yet this does not occur in other hypothalamic areas thus suggesting that 5-HT promotes the genesis of 5-HTergic neurons specifically in the PVO that will be spread along the brain ventricles (Pérez et al. 2013). The PVO also displays a prominent population of dopaminergic cells (Yamamoto et al. 2010) but none show double phenotype (Sallinen et al. 2009). The PVO seems to take part in the ascending dopaminergic midbrain system of fish which integrates three subsystems in tetrapods such as the mesolimbic (reward response), mesocortical (learning and memory) and mesostriatal (sensorimotor) (Rink and Wullimann 2001; Yamamoto et al. 2010) systems. Therefore, hypothalamic 5-HT could be involved in the regulation of several behavioural responses. In fact, 5-HT is a well-known anxiolytic agent in vertebrates which also regulates feeding behaviour in fish (Rubio et al. 2006; Ceinos et al. 2008; Nowicki et al. 2014; Soares et al. 2018; Ziegler et al. 2020).

*Tph2*-expressing neurons first appear in two neuronal populations of the pretectal area, such as the dorsal and ventral part of the periventricular pretectal nucleus (PPd and PPv). Both populations surround the dorsal and ventral aspects of the fasciculus retroflexus (FR) placed in the most dorsal pole of the third ventricle, respectively. These populations correspond to the “area 1” of 5-HT immunoreactive neurons previously described by Frankenhuis-van den Heuvel and Niewenhuys (1984). These 5-HT neurons have no homologues in tetrapod species suggesting that it is a fish-specific characteristic. In fact, pretectal serotonergic neurons have been reported in most studies on fish (reviewed in Lillesaar 2011; Gaspar and Lillesaar 2012) with the exception of flatfish Senegalese sole (Rodríguez-Gómez et al. 2000). It has been suggested in tilapia that periventricular pretectal nucleus conveys sensory information from visual and lateral line pathways into the cerebellum (Xue et al. 2007). The presence of tph2-expressing neurons in the VM is more controversial as serotonergic studies using specific antibodies in many species have not reported 5-HT immunoreactive neurons in the VM (Lillesaar 2011). However, studies in trout showed some immunoreactive neurons stretching dorsally along the median thalamic line within the “area 2” (Fig. 5c in Frankenhuis-van den Heuvel and Niewenhuys 1984). Accordingly, studies in lamprey reported a *tph1*-expressing neuronal population in the thalamus at the same level in which pretectal 5-HT neurons were located (Fig. 1F in Rincón-Camacho et al. 2016). *Tph2*-expressing neurons were also found downstream in the raphe and reticular formation of the hindbrain of the rainbow trout CNS. A conspicuous population of *tph2*-expressing neurons in the SRa was detected yet scattered *tph2*-labelled neurons in the more caudal IRa were also observed. Serotonergic neurons in the raphe are characterized by the expression of the ETS-domain transcription factor-encoding gen *pet1* which is essential for the development of the brainstem 5-HT system (Lillesaar et al. 2007). Using a transgenic zebrafish overexpressing green fluorescent protein (GFP) under the control of *pet1* proximal promoter, Lillesaar et al. (2009) initially characterized two serotonergic populations in the SRa, such as the 5-HT neurons located in the midline of the hindbrain and a second overlooked serotonergic population found in the ventrolateral hindbrain of zebrafish. The latter population projects to the migrated nuclei of the posterior tuberculum. Tracing studies combined with *pet1*-directed GFP expression have demonstrated that cells in and along the SRa midline projecting to the hypothalamus tend to be more ventrally localized and exhibit larger neuronal bodies than those projecting to olfactory bulbs and the telencephalon. Projections coming from dorsal and ventral populations are arranged into different clusters thus revealing some functional organization within the SRa (Lillesaar et al. 2009). Morphological studies have also suggested a functional subdivision of the SRa in plainfin midshipman (*Porichthys notatus*) (Timothy and Forlano 2020). Our in situ hybridization experiments in rainbow trout only detected the midline serotonergic population. However, two serotonergic subpopulations, dorsal and ventral, could be differentiated in the most rostral midline region of the SRa thus suggesting some kind of functional organization, but this assumption is only based on morphology and location of the tph2-expressing neuronal bodies in the SRa. Such neuronal population would correspond to the “area 5” from Frankenhuis-van den Heuvel and Niewenhuys (1984). Our expression studies were unable to locate the 5-HT ventrolateral neurons described in the zebrafish and stickleback hindbrain (Ekström and Van Veen 1984) yet 5-HT immunoreactive neurons were described in the dorsolateral position to the fasciculus longitudinalis medialis, at the level of the superior raphe (“area 6” in Frankenhuis-van den Heuvel and Niewenhuys 1984). These neurons could correspond to those described in zebrafish and stickleback yet could also be a distinct population of pseudoserotonergic cells of the trout CNS. Alternatively, it cannot be discarded that other rainbow trout *tph1* paralogues were expressed in this location.

Serotonergic neurons in the IRa of the brainstem of trout lie more caudally in the ventral region of the midline. The presence of true serotonergic neurons in the IRa is a constant characteristic of the vertebrate brain (Lillesaar 2011; Gaspar and Lillesaar 2012; Timothy and Forlano 2020). Even in lamprey two rhombencephalic populations (isthmic and caudal) have been characterized presumably as homologues of the raphe nuclei (Barreiro-Iglesias et al. 2008; Cornide-Petronio et al. 2013). Studies in mammalian species demonstrated that SRa and LRa 5-HT neurons project to the rostral and caudal regions, respectively. This polarization in the projection patterns was also verified in zebrafish (McLean and Fetcho 2004). However, studies by Lillesaar et al. (2009) using pet1:eGFP transgenic line zebrafish showed that a minor population of 5-HT neurons in the SRa project caudally into the hindbrain but not further than the spinal cord.

In summary, the expression distribution of one *tph1* paralogue (out of three) was reported, such as the *tph1b2* and *tph2* gene in rainbow trout. The *tph1* and *tph2*-expression distribution is compared to the reported 5-HT immunoreactive neurons previously described thus showing the true serotonergic territories in the trout brain. Results show that segregated expression for both isoforms primarily match immunocytochemical studies but some relevant variations were found to be predominantly localized in the ventral thalamus, hypothalamic lateral recess and rostral hindbrain populations. Our research provides further insight into the very few and restricted studies addressing the serotonergic marker expression to characterize the true serotonergic brain territories.

## Supplementary Information

Below is the link to the electronic supplementary material.

Supplementary file 1 Figure S1. Phylogeny of tph proteins. Multiple sequence alignments of tph amino acid sequences for all taxons (A), fish species (B) and salmonid species (C) were generated using ClustalX 2.1 and the evolutionary history was inferred by using the Maximum Likelihood method based on the JTT matrix-based model [1]. The tree with the highest log likelihood is shown. The percentage of trees in which the associated taxa clustered together is shown next to the branches. Initial tree(s) for the heuristic search were obtained automatically by applying Neighbor-Join and BioNJ algorithms to a matrix of pairwise distances estimated using a JTT model, and then selecting the topology with superior log likelihood value. The analysis involved 111 amino acid sequences. All positions with less than 95% site coverage were eliminated. That is, fewer than 5% alignment gaps, missing data, and ambiguous bases were allowed at any position. There were a total of 362 positions in the final dataset. Evolutionary analyses were conducted in MEGA7. (PDF 102 KB)

## Data Availability

Data are available on reasonable request.

## Code availability

No applicable.

## Declarations

### Studies involving humans and/or animals

No applicable.
