# Supplementary figures and images for: Distribution of two isoforms of tryptophan hydroxylase in the brain of rainbow trout (Oncorhynchus mykiss). An in situ hybridization study
Source: Brain Struct Funct. 2021 Jul 2;226(7):2265–78. doi: 10.1007/s00429-021-02322-8 (PMC8354878; doi:10.1007/s00429-021-02322-8)

A

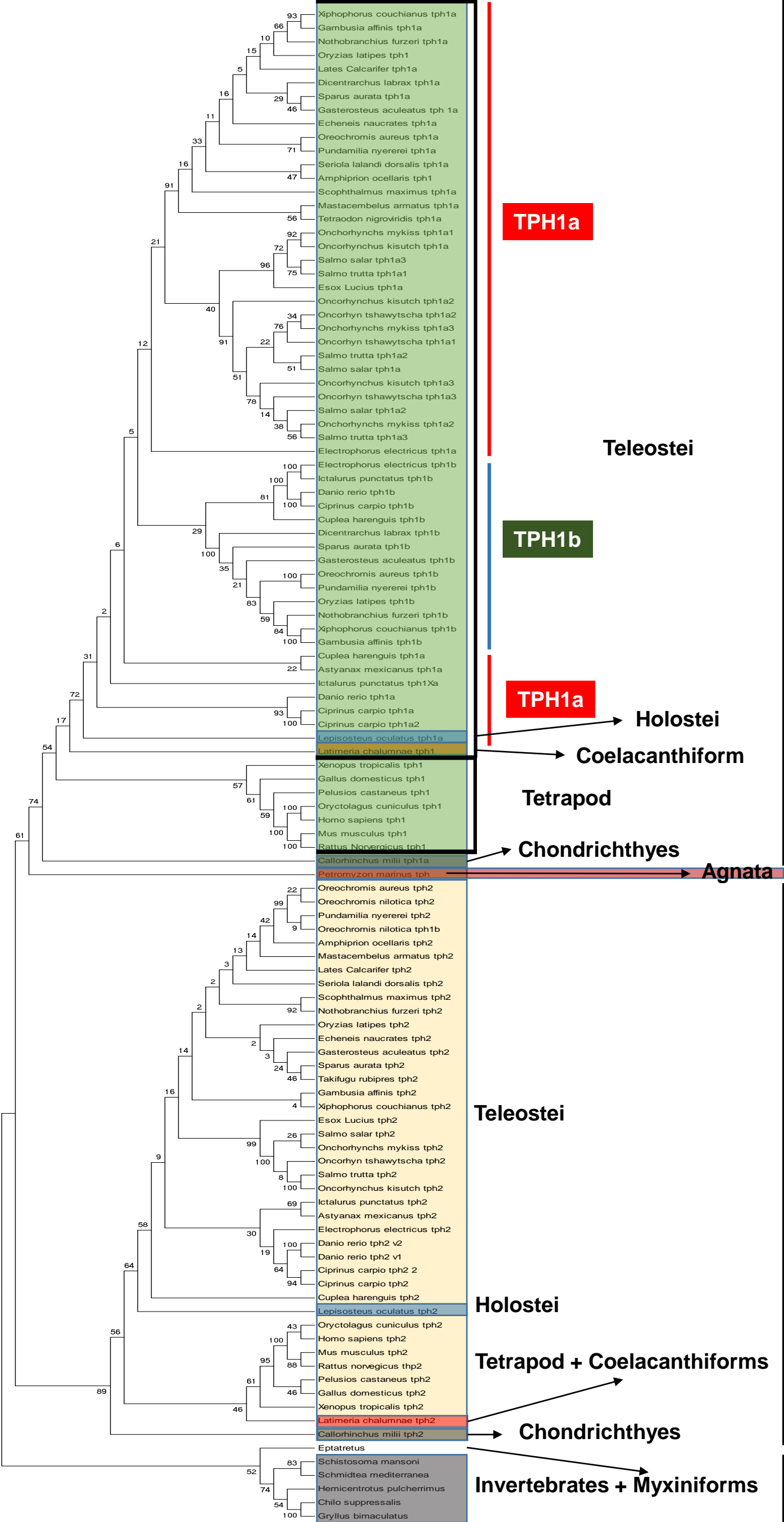

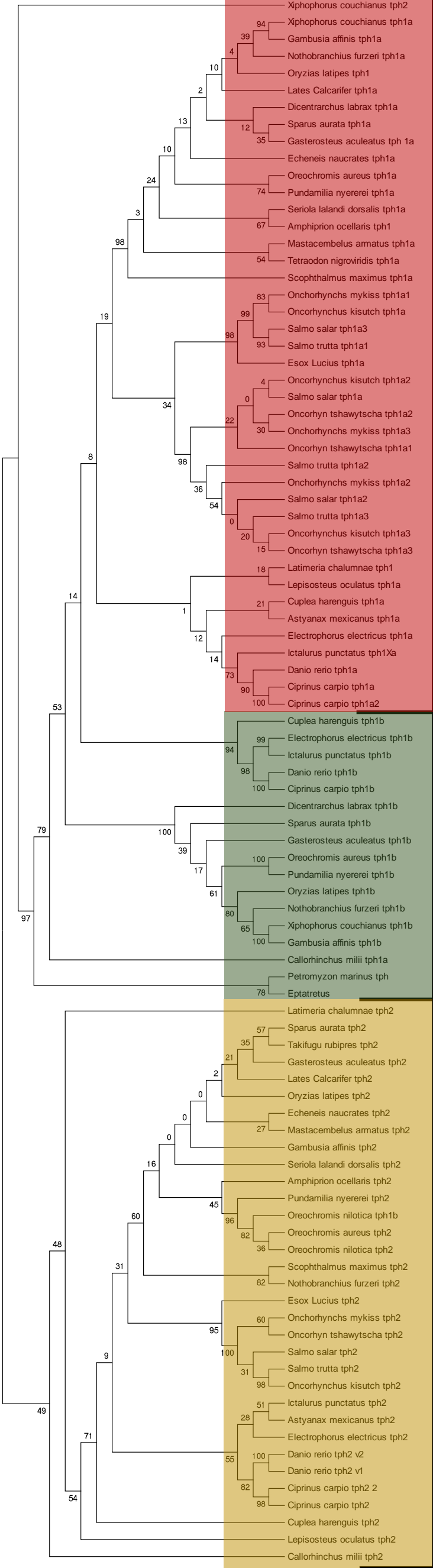

TPH1a

TPH1

TPH1b

TPH2

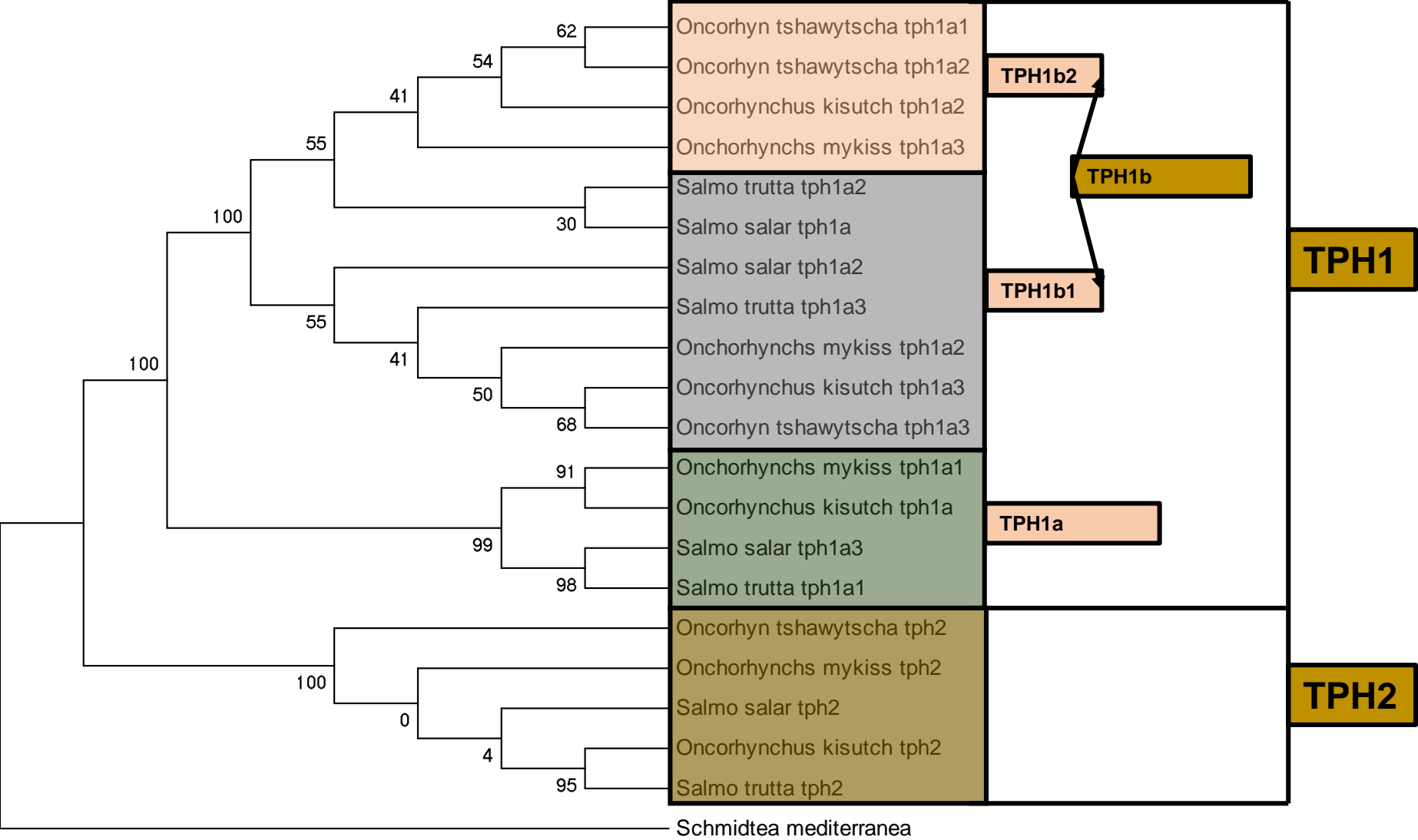

Supplement: Supplementary file 1 — Figure S1. Phylogeny of tph proteins. Multiple sequence alignments of tph amino acid sequences for all taxons (A), fish species (B) and salmonid species (C) were generated using ClustalX 2.1 and the evolutionary history was inferred by using the Maximum Likelihood method based on the JTT matrix-based model [1]. The tree with the highest log likelihood is shown. The percentage of trees in which the associated taxa clustered together is shown next to the branches. Initial tree(s) for the heuristic search were obtained automatically by applying Neighbor-Join and BioNJ algorithms to a matrix of pairwise distances estimated using a JTT model, and then selecting the topology with superior log likelihood value. The analysis involved 111 amino acid sequences. All positions with less than 95% site coverage were eliminated. That is, fewer than 5% alignment gaps, missing data, and ambiguous bases were allowed at any position. There were a total of 362 positions in the final dataset. Evolutionary analyses were conducted in MEGA7. (PDF 102 KB) [file 429_2021_2322_MOESM1_ESM.pdf]
